# Sexual minority health inequalities — why are we unable to do more?

**DOI:** 10.1186/s44263-024-00066-1

**Published:** 2024-06-03

**Authors:** Amal R. Khanolkar

**Affiliations:** Department of Population Health Sciences, King’s College London, Addison House, Guy’s Campus, London, SE1 1UL UK

Sexual and gender minority (SGM) individuals report worse mental and physical health compared to heterosexual and cisgender peers. Growing evidence, positive legislation and interventions have helped reduce sexual and gender-related discrimination. Nevertheless, a lack of SGM relevant data and research infrastructure impedes development in this research area.

## Health inequalities in SGM groups

Recent decades have witnessed considerable advancement in sexual and gender minority (SGM) legal rights and progressive changes in societal acceptance and understanding of sexuality in many countries. Despite these advancements, SGM individuals (those who self-identify as non-heterosexual, lesbian, gay, bisexual, transgender, queer [LGBTQ +] and/or gender minority) continue to report poorer mental health compared to their cisgender and heterosexual peers [1]. SGM individuals report substantially higher levels of mental health problems (like depression, anxiety, stress, PTSD) and eating disorders, self-harm and suicidality amongst other conditions [1]. These mental health conditions are often 2–5 times higher in SGM populations compared to heterosexual peers, and reported in multiple study populations globally using national surveys, cohorts, electronic health records and national registers, and across all life stages. There are also differences in risk for poorer mental health between SGM subgroups, for example bisexual and transgender individuals report higher levels of depression, anxiety, PTSD and suicidality compared to gay and lesbian peers [1]. These health inequalities are not restricted to mental health. SGM individuals are also at increased risk of many physical health conditions (like heart disease, cancer, asthma, diabetes, back pain) [2, 3]. Recent and limited evidence also indicate higher risk for mental and physical health comorbidities including multiple long-term conditions (or multimorbidity) in SGM individuals compared to heterosexual and cisgender peers [2]. SGM individuals also report higher levels of health-risk behaviours (like drug and alcohol misuse and risky sex), often suggested to be coping mechanisms to deal with discrimination and living with substantially worse mental health [4]. Adverse mental health in SGM individuals is largely attributed to experiences of chronic and acute stressors associated with stigma and discrimination related to SGM identities [5, 6]. These minority-identity-related stressors are over and above regular stressors experienced by people in the general population [6].

## Intersectionality of SGM and other minority identities

Research has also focused on SGM individuals with intersectional minority identities (most often ethnic and/or faith minority individuals who identify as SGM) [7]. These individuals encounter a different set of challenges compared to White-SGM peers including ethnic-specific expectations (like cultural obligations including educational, familial and community expectations, rejection by their communities), which can be challenging to navigate in different life stages. They also face racism, bullying and unconscious bias related to both identities (independently and together; a ‘double burden’), experiences they often encounter in their ethnic communities, the general population and in multiple locations (like residential neighbourhoods, workplaces) [8]. The intersectionality framework theory postulates adverse health in individuals with ≥ 2 minority identities is due to the intersection between multiple ‘marginalised minority identities’ associated with different forms of and higher levels of discrimination within set social hierarchies resulting in multiple levels of inequality [9].

The overwhelming research on health in SGM groups originates from the USA. The UK (and to some extent many European countries) have largely conducted research on prevalence of health conditions in SGM groups with a few limited studies of longitudinal design exploring pathways and mechanisms to health inequalities.

## SGM health research and the situation in the UK

The UK has witnessed significant and positive steps in legal rights for SGM groups including the Equality Act 2010, civil partnership and marriage, adoption and family rights. The British society has also witnessed greater acceptance and understanding of sexuality and SGM rights, correlating with a greater number of individuals, especially from younger generations, identifying as SGM (for example 6% of individuals < 35 years identified as non-heterosexual in 2014 increasing to 17% in 2022). Given the positive change experienced by society in greater acceptance and legal rights for SGM individuals, inequalities in health between heterosexual and SGM individuals should be decreasing. However, this is not the case globally and in the UK [1, 2]. Several recent UK studies on younger individuals including adolescents from contemporary cohorts continue to report worse mental and physical health and higher levels of health-risk behaviours compared to heterosexual peers [4]. A combination of factors might potentially explain the persisting SGM-related inequalities. SGM individuals continue to face identity-related stigma and discrimination across all ages and in diverse environments despite legislation trying to reduce such discrimination. There could be relative stability of minority-related stressors over time. Current legislation needs to be further developed to protect SGM individuals. Sexuality-based research has not yet disentangled the complex pathways between sexuality and health that could inform much needed public health policy to reduce inequalities, especially policies and interventions that directly address minority-related stressors. However, to conduct such research, the appropriate infrastructure is required, which is currently lacking in the UK [10]. Nevertheless, the UK remains a global leader in conducting population-based cohort studies and a multitude of surveys. Many of these resources do not collect sexual identity information and at times are not available to researchers without additional clearances, or when collected appropriately, it is in surveys that by design collect limited information and preclude longitudinal follow-up of the same individuals. More recent and larger studies like the UK Biobank and *Our Future Health* however have included questions on sexual/gender identities. Further, the more recent UK research studies like Millennium Cohort Study, Avon Longitudinal Study of Parents and Children and Next Steps collect data on sexual/gender identities and have contributed substantially to the existing research body on sexuality-related health in younger generations [4, 10]. It is important to note that despite relatively large numbers of sexual minority individuals in these studies, the numbers are often insufficient to examine differences between sexual minority subgroups and across different intersectional identities, mostly due to lack of statistical power [4]. This frequently results in combining or ‘lumping’ sexual minority subgroups together often criticised as inadequate as it does not help in better understanding of nuanced differences between distinct groups and has implications for policy. The knowledge gaps in health of older SGM individuals are more apparent due to the paucity of data in older cohorts. Most research on older SGM individuals relies on data from surveys like the General Practice Patient Survey [2, 11]. These are informative as a ‘first step’ in examining the differences in prevalence of health conditions and access to healthcare and designing more comprehensive research questions and projects. The situation is further compounded by the fact that sexual identity is rarely captured in routinely collected health data in general practices and hospitals as often this information is considered to be too sensitive.

## Research infrastructure: data-driven solutions

The main barrier to continuing more detailed and substantive research on sexuality and health is simply the lack of appropriate data in the UK. SGM individuals experience vastly different lived experiences throughout their lives compared to cisgender and heterosexual peers. These experiences impact and shape their encounters and interactions across all life dimensions (life stages like childhood, adolescence, adulthood, older age; environments like school, work, places of worship, neighbourhoods they reside and work in; accessing and using healthcare services; and so on). SGM individuals also experience unique situations like ‘coming out’, internalised stigma and rejection by families, friends, society, etc., which are established risk factors for poorer health. Thus, we need to go beyond just including SGM individuals in research studies and surveys. We need to collect *SGM relevant data* in addition to commonly collected health and social data. The lack of appropriate research infrastructure is both surprising and concerning as persistent sexuality-related health inequalities are well known and the UK does not lack the expertise or capability to set up the necessary infrastructure. The limited funding available for addressing SGM-based research is well recognised [10]. This lack of funding not only impacts the setting up of required infrastructure but also it affects marginalised scholars who are more likely to work in this field [10]. Thus, many researchers rely on the existing (often publicly) available secondary data, which is insufficient for the reasons described above.

## Looking ahead

There must be a priority shift in how we address and conduct SGM-based research more generally and health inequalities research in particular. It is reassuring that some of the latest studies set up on health include routine questions on sexual and gender identities. This should be implemented across all existing and future research studies and surveys. There should be a concerted effort to collect SGM relevant data (including lived experiences) both in existing research studies and surveys but also by initiating specific cohort studies focused on SGM health. We must ensure adequate representation of all SGM subgroups and intersectional identities, not only for better statistical power purposes but also to understand nuanced differences between the subgroups and various intersectional identities. This can be addressed by oversampling SGM individuals in studies (already a routine practice for ethnic minority and socioeconomically disadvantaged groups) and initiating SGM-specific cohort and survey studies. Further, incentives should be provided to collect data on sexual and gender identities in primary and secondary healthcare settings (in most instances, this would include one additional question in preexisting forms that can be voluntary).

Given the existing and robust research infrastructure for population health, appropriate initiatives in combination with adequate funding can enable the UK to take a global lead in SGM health research and reduce health inequalities.

## Data Availability

No datasets were generated or analysed during the current study.

## Abbreviations

SGM: Sexual and gender minority
LGBTQ +: Lesbian, gay, bisexual, transgender, and queer
PTSD: Post-traumatic stress disorder
USA: United States of America
UK: United Kingdom
